# Exploring the Dynamic Interaction Between Pituitary Neuroendocrine Tumors (Pit-NETs) Cells and Their Angiogenic Microenvironment by Using the MIB1 Labeling Index, VEGF Expression and Digital Image Analysis

**DOI:** 10.3390/cimb48010027

**Published:** 2025-12-25

**Authors:** Mihaela Cozma, Anca Maria Cimpean, Mihail Parnov, Ana Silvia Corlan, Silvia Stratulat, Paula Fala, Eugen Melnic

**Affiliations:** 1 Department of Pathology, Nicolae Testemitanu State University of Medicine and Pharmacy, MD 2004 Chisinau, Moldova; mihaelacerlat@gmail.com (M.C.); mihail.parnov@usmf.md (M.P.); eugen.melnic@usmf.md (E.M.); 2 Timofei Mosneaga, Republican Clinical Hospital Chisinau, MD 2004 Chisinau, Moldova; 3 Department of Microscopic Morphology/Histology, “Victor Babeș” University of Medicine and Pharmacy Timisoara, Eftimie Murgu Square No 2, 300041 Timisoara, Romania; 4 Center of Expertise for Rare Vascular Disease in Children, Emergency Hospital for Children Louis Turcanu, 300011 Timisoara, Romania; 5 Center for Genomic Research, GENOMICA, “Victor Babeș” University of Medicine and Pharmacy Timisoara, Eftimie Murgu Square No 2, 300041 Timisoara, Romania; 6 Research Center for Pharmaco-Toxicological Evaluation, “Victor Babeș” University of Medicine and Pharmacy, Eftimie Murgu Square No 2, 300041 Timisoara, Romania; 7 Department of Internal Medicine II, Discipline of Endocrinology, “Victor Babeș” University of Medicine and Pharmacy Timisoara, 300041 Timisoara, Romania; 8 Department of Biochemistry, Nicolae Testemitanu State University of Medicine and Pharmacy, MD 2004 Chisinau, Moldova; silvia.stratulat@usmf.md; 9 Department of Neurology 1, Nicolae Testemitanu State University of Medicine and Pharmacy, MD 2004 Chisinau, Moldova; paula.fala@usmf.md

**Keywords:** PitNETs, endothelial cells, tumor cells, proliferation, digital image analysis, QuPath

## Abstract

One controversial issue in pituitary pathology is the simultaneous proliferation of PitNETs and endothelial cells. No previous studies have compared the MIB1 Labeling Index (MIB1 LI) of PitNETs and stromal endothelial compartments and its connection with VEGF protein and gene expression. Simultaneous PitNETs proliferation index assessment in tumor and endothelial cells is related to VEGF protein and gene expression, and by using the automated QuPath platform for digital image analysis (DIA), it can be determined whether this dual proliferation specifically characterizes certain PitNETs subtypes. A total of 109 PitNETs were immunostained for endothelial cells (CD34) and proliferation (MIB1). VEGF was assessed by using IHC and RNA scopes. QuPath_DIA measured hormone-dependent MIB1 nuclear expression in tumor and stromal endothelial cells. MIB1 LI correlated with VEGF_mRNA and protein expression. PRL-secreting and non-functioning PitNETs had a high MIB1 LI in stromal endothelial cells. MIB1-positive tumor cell (%MIB1 LI.T) and endothelial cell (%MIB1 LI.E) percentages were substantially correlated (*p* = 0.01). The profiles of VEGF and hormones significantly and heterogeneously impact the MIB1-LI of tumor and endothelial cells. Tumor–endothelial cell proliferative interaction is specific to PRL-secreting and non-functioning PitNETs. These findings suggest that digital analysis of MIB1 and VEGF expression may serve as a valuable tool for risk stratification in PitNETs.

## 1. Introduction

PitNETs, or pituitary neuroendocrine tumors, are tumors arising from the hormone-secreting cells of the anterior pituitary gland, previously referred to as pituitary adenomas. Although generally benign (non-cancerous), they may induce considerable health complications by interfering with hormone synthesis or exerting pressure on adjacent structures. Pituitary adenomas and pituitary carcinomas are categorized as neuroendocrine tumors, more precisely termed pituitary neuroendocrine tumors (PitNETs). The WHO classification of endocrine and neuroendocrine tumors (2025) and the WHO classification of CNS tumors (2021), 5th edition, include “PitNET” alongside pituitary adenoma (instead of replacing it) [1,2]. At present, until there is extensive acceptance and acknowledgment of this new terminology, it seems prudent to continue using the term adenoma, or at the absolute least, to mention it in parentheses. PitNETs are classified according to the specific hormone they influence and their degree of invasiveness [1,2]. According to their size, they are classified as microadenomas (<10 mm in size) or macroadenomas (>10 mm in size). Based on their secretory profile, PitNETs are found to be secretory (about 65% as prolactin-secreting −50%, growth hormone-secreting −10%, adrenocorticotropin-secreting −6%, thyrotropin-secreting −1% and mixed) and non-secretory (or non-functional, about 35%). Hormone-specific types of PitNETs are currently included in subgroups according to PIT1, SF1 and TPIT transcription factor expression. Non-functional PitNETs (NFPitNETs) frequently have parasellar or suprasellar extension, and complete tumor removal is usually not feasible, leading to persistent tumor development or recurrence. Functional PitNETs can be readily monitored for recurrence via hormonal biomarkers; however, there is no analogous measure for predicting recurrence of NFPitNETs, hence hindering early detection and prompt intervention. Consequently, it is essential to find predictive biomarkers for patient monitoring and therapy targeting. The correlation of already known data from the tumor compartment to vascular and cellular stromal components could be an alternative to finding new biomarkers with potential prognostic impact for NFPitNETs.

Currently, in a relatively new PitNET classification, the invasiveness is mostly exclusively correlated to the behavior of PitNET cells, while the stromal compartment is totally neglected [3,4]. Few papers related to PitNET stroma are available in the literature. By searching on PubMed using search words such as “pituitary adenoma stroma”, about 90 were found, published between 1970 and the present [5]. More surprisingly, identification of PitNETs’ proliferating stromal blood vessels (by using CD34/MIB1 double immunostaining) was extremely rare; just nine papers in the field have been previously published related to this subject [6].

The vascular density observed in both benign and aggressive pituitary neuroendocrine tumors (PitNETs) is inferior to that found in the normal hypophysis [7]. Beyond PRL secretion, the scientific literature provides scant evidence indicating a decrease in blood vessel density in various other forms of PitNETs. The low blood vessel density observed in PRL secreting PitNETs could be related to the existence of a 16 kDa fragment of prolactin, which exerts inhibitory influences on tumor angiogenic growth factors and endothelial cells. Consequently, the occurrence of angiogenesis in PitNETs, lacking a detailed study of angiogenic mechanisms and the involved pathways, raises several controversies [8]. Recent findings suggest that PitNETs exhibit a reduced capillary size compared to normal pituitary tissue. Furthermore, a significant correlation exists between this particular type of blood vessel and nestin expression [9,10].

Previous research on PitNETs has predominantly focused on their morphological and ultrastructural changes [11,12], while only a few studies have focused on their immunohistochemical or molecular features [13]. The paper published by Sav et al. [13] critically reported several controversies regarding PitNET angiogenesis related to the angiogenic growth factors VEGF, COX2 or Pituitary Tumor Transforming Gene (PTTG) as well as to PitNET microvessel density (MVD) assessed by using CD34 immunohistochemistry. The authors reported that the colocalization of PTTG and VEGF in the same PitNET cells correlated to a high CD34_MVD, especially in growth hormone (GH)-secreting PitNETs. The same paper also reported the usefulness of Ki 67 proliferation as an immunohistochemistry marker in the evaluation of PitNETs invasiveness and aggressiveness, but these data were restricted to PitNETs proliferation and did not include endothelial proliferation from the PitNETs stroma [13].

The presence of blood vessel heterogeneity in PitNETs was first suggested in PRL secretion by Schechter et al. in 1988 [14] following an electron microscopic analysis of endothelial and perivascular cells. He observed the presence of unusual capillaries surrounded by multiple layers of perivascular cells in PRL secreting PitNETs, contrasting with the standard fenestrated capillaries characteristic of the normal pituitary gland. Other variants of PitNETs were not investigated regarding their vascular morphology and immunophenotypic characteristics. Moreover, the impact of tumor vasculature heterogeneity on the clinical characteristics of PitNETs remains undocumented in the existing literature.

Vascular endothelial cell proliferation is one of the early steps of tumor neoangiogenesis and it signals the presence of endothelial cell activation followed by the formation of new blood vessels. Once the PitNET vascular network is developed and new blood vessels are recruited by PitNETs, tumor cell aggressiveness seems to increase. Tumor cell aggressiveness includes an increase in both tumor cell proliferation and their ability to spread locally and to invade surrounding tissue, leading to clinical and radiological signs of compression. A total of 0.2% of PitNETs may present metastasis and are classified as pituitary carcinomas [15]. In such PitNETs, vascular invasion is the most common way for metastatic cells to spread. Non-functioning PitNETs exhibit vasculogenic mimicry (VM) as their main mechanism of invasion [16]. According to data from the literature, cell proliferation and angiogenesis play a limited role in non-functioning PitNETs. However, our understanding of their role may be due to the lack of standardized, automated methods to quantify the expression of nuclear proliferation markers and the use of subjective interpretation of IHC results related to density a neo angiogenesis subjective criterion for assessing immunohistochemistry results. A VEGF scoring of +1, +2, or +3 refers to VEGF-positive PitNET cell density but does not consider the intensity of expression. Low or high VEGF expression can be used as a subjective immunohistochemistry marker of PitNETs [17]. The MIB-1 Labeling Index (LI) is a pathology measure that uses Ki-67 staining (MIB-1 antibody) to quantify the percentage of actively dividing (proliferating) cells in a tissue sample. MIB1 assessment is usually performed via microscopic analysis of positive nuclei using manual counting [18] and reporting as the percentage of positive nuclei per 1000 cells or 10,000 cells subjectively observed in tumor cells, but not in endothelial cells.

Considering previously published data, we aim to perform a comparative evaluation of tumor and endothelial cell proliferation in PitNETs using double immunohistochemistry for CD34/MIB1 (CD34 to identify endothelial cells and MIB1 to highlight proliferative cells). For an accurate immunohistochemistry assessment, we performed digital image analysis (QuPath analysis), enabling us to observe cell density as well as the intensity of immunohistochemical staining. The proliferative index of both tumor and endothelial cells will be correlated to VEGF expression specifically for each PitNET subtype.

## 2. Materials and Methods

Study Design. The present study is a single-center retrospective study of 109 patients with PitNETs admitted to the Neurosurgery Department of Timotei Mosneaga Republican Hospital between 2009 and 2024 (15 years) for surgical therapy of PitNETs. PitNET removal was performed via a transsphenoidal surgical procedure, and tissue biopsies were immediately preserved via immersion in 10% buffered formalin and sent to the Pathology Department of the same hospital. Normal pituitary tissues (*n* = 10) were harvested during autopsy of patients who died due to other reasons which usually do not affect normal pituitary gland architecture. Formalin-fixed paraffin-embedded (FFPE) tissue blocks were selected based on specific inclusion and exclusion criteria. Inclusion criteria were as follows: (1) age between 18 and 78 years old; (2) no disease associated with pituitary involvement; (3) male/female ratio of 1:1; (4) clinical and pathologic diagnosis of PitNETs; (5) no other associated benign/malignant tumors; (6) no previous antiangiogenic/antivascular therapies; (7) no previous chemotherapy; (8) no history of Multiple Endocrine Neoplasia; (9) no oral antidiabetic drugs (metformin mainly due to its antiangiogenic and antiproliferative effects); and (10) positive informed consent from patients to use their tissue for research purposes. Exclusion criteria targeted all patients who did not meet the inclusion criteria or those with a diagnosis other than PitNETs. The study was conducted in accordance with the Declaration of Helsinki and approved by the Ethics Committee of both Victor Babes University of Medicine and Pharmacy Timisoara Romania and Nicolae Testemitanu State University of Medicine and Pharmacy, Chisinau, Moldova. Informed consent was obtained from all subjects involved in the study, ensuring patient agreement to the use of their tissue fragments for research purposes and respecting their personal data.

Primary processing of tissue biopsies. A total of 109 specimens were collected from patients diagnosed with PitNETs, all of whom had undergone prior surgical treatment. The biopsies underwent immersion in a 10% buffered formalin solution for a duration of 48 h, subsequently being embedded in paraffin as scaffold to protect the tissue sections. From each paraffin block, three 3 μm serial sections were accurately obtained. The assessment of tissue pathology was performed on slides that were stained using routine hematoxylin and eosin (H&E) followed by microscopic examination for histopathologic diagnosis confirmation and evaluation of tissue section quality for future immunohistochemistry analysis (integrity of tissue section, lack of artifacts which can negatively influence immunohistochemistry results and RNAscope results).

Immunohistochemistry. Before all other specific immunohistochemical stainings, adequate tissue quality was confirmed according to positive immunostaining for Vimentin, clone V9 (Leica Microsystems, Milton Keynes, UK) in the connective tissue from PitNETs. PitNET classification according to different hormone subtypes was achieved through immunohistochemistry analysis and by using antibodies specific to each of the hormones produced by the pituitary gland, encompassing growth hormone (GH, polyclonal, rabbit anti-human, ready to use), prolactin (PRL, polyclonal, rabbit anti-human, ready to use), adrenocorticotroph hormone (ACTH, polyclonal, rabbit anti-human, ready to use), thyroid-stimulating hormone (TSH, polyclonal, rabbit anti-human, ready to use), follicle-stimulating hormone (FSH, polyclonal, rabbit anti-human, ready to use) and luteinizing hormone (LH, polyclonal, rabbit anti-human, ready to use). All primary antibodies specific to pituitary hormones were generously supplied by Dako Cytomation (Carpinteria, CA, USA) and were ready to use (RTU), eliminating the need for dilution. The single immunostain was performed by using the Bond Polymer Refine Detection System from Leica Biosystems (Milton Keynes, UK) which includes secondary antibodies and hematoxylin for the counterstain. We replaced diaminobenzidine with methyl green as the chromogen due to the increase in contrast between the cytoplasm and nuclei of endothelial cells. Endothelial cells were identified through incubation for 30 min at room temperature with anti-CD34 antibodies (monoclonal mouse anti human, clone QBEnd10, RTU, Dako Cytomation, Carpinteria USA, methyl green as chromogen), while the proliferating nuclei were distinguished using anti-MIB1 antibodies (monoclonal mouse anti human, clone MIB1, RTU, brown with diaminobenzidine, Dako Cytomation, Carpinteria, USA). The double-stain method was employed as a visualization technique to distinctly identify CD34-positive endothelial cells in green and MIB1-positive endothelial cell nuclei in brown. Counterstaining was performed through the application of a modified variant of Lillie Hematoxylin. The comprehensive immunohistochemical protocol was carried out utilizing a Max Bond Autostainer (Leica Microsystems, Milton Keynes, UK). VEGF (concentrated mouse anti-human monoclonal antibody, clone VG1, dilution 1:25, 30 min incubation time at room temperature) was supplied by the same vendor as the antibodies for pituitary hormones. Positive controls included tubular structures of the kidney. Immunostaining was evaluated as positive when over 10% of tumor cells exhibited labeling. The immunolabelling index was classified as 0 (negative), 1+ (10–30% of cells), 2+ (31–50% of cells), or 3+ (more than 50% of cells). Double immunostaining for CD34 and MIB1 allowed us to identify proliferating blood vessels from normal pituitary tissue and various PitNET subtypes together with proliferating tumor cells. Both proliferating statuses were evaluated in the same microscopic area, and this allowed us to compare MIB1 LI between tumor and stromal compartments. We identified proliferative blood vessels (CD34+/MIB1+), varying in number between 6 and 20, amongst different subtypes of PitNETs; we marked them by using digital tools and automatically assessed MIB1-positive nuclei according to digital image analysis software, version 0.6.0.

RNAscope assessment of VEGF mRNA on PitNET tissue specimens. RNAscope, an in situ *hybridization* approach for RNA on formalin-fixed paraffin-embedded (FFPE) specimens, was used to validate the immunohistochemistry results for VEGF by assessing ribonucleic acid. We observed a single molecule in individual cells on specimens that were paraffin-embedded and formalin-fixed. Briefly, the tissue sections underwent pretreatment to permeabilize cells. Double Z probes were hybridized to target RNA molecules, followed by amplification of the hybridization signals through sequential hybridization of amplifiers and label probes. Ultimately, the RNA molecules were visualized as brown signals using chromogen 3,3′-diaminobenzidine. Amplification of VEGF-mRNA was rated on a scale from 0 (no staining or less than 1 point for every 10 cells, ×400 magnification) to 4 (>10 points/cell, >10% of positive cells with clusters of points visible at ×400 magnification).

**Figure 1:**
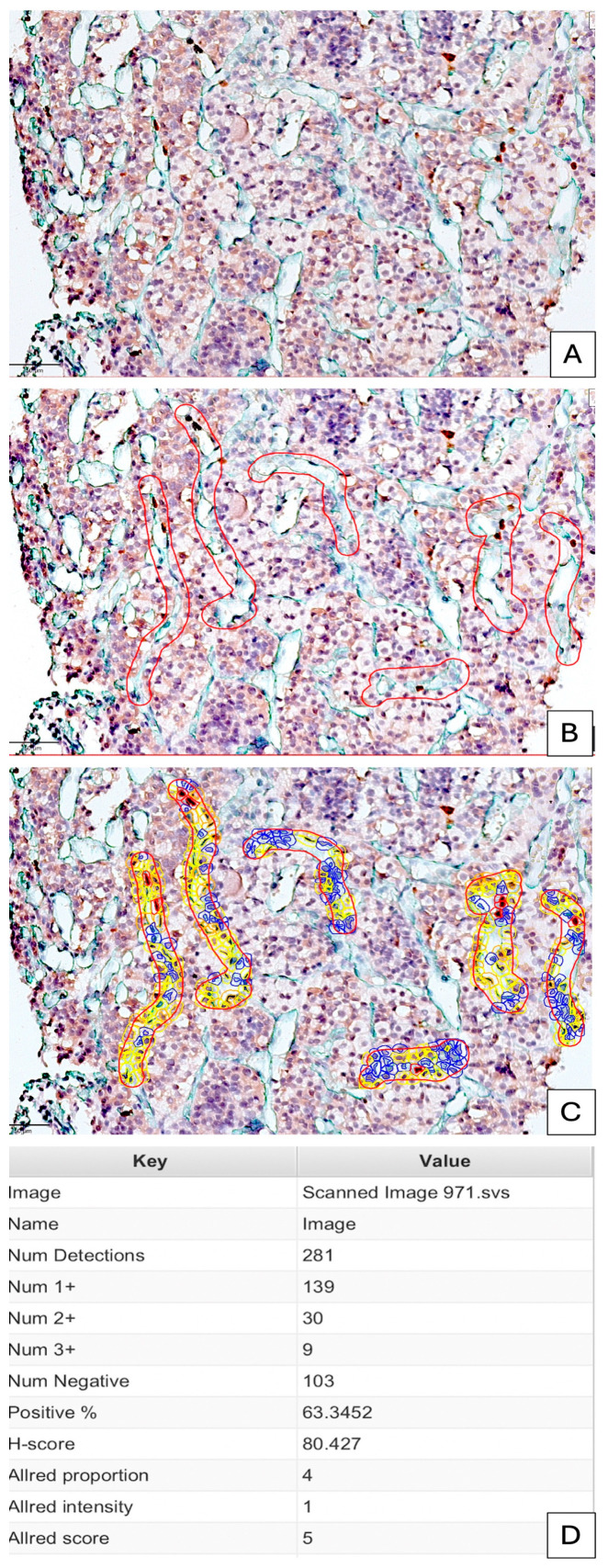
Conventional microscopy (**A**) versus QuPath-based (**B**–**D**) comparative assessment of MIB1 LI of normal human pituitary vascularization (**A**) and endothelial cell proliferative status. By using a brush digital tool, red blood vessel lined by proliferative endothelial cells are marked (**B**). By analyzing positive cells from the QuPath menu, we highlighted only nuclear-positive MIB1 structures, evaluated separately according to density and intensity (**C**) as negative (**C**, blue), low (**C**, yellow), medium (**C**, orange) and high (**C**, red). All detection measurements were automatically calculated and reported as shown in the figure (**D**). The Allred score, H-score and percentage of positive nuclei for MIB1 represent the main parameters for the automated evaluation of the proliferative index.

Digital Image Analysis. IHC samples were scanned using a Grundium OCUS 20 Microscope (Grundium, Tampere, Finland) and stored in the Case Center Slide Library as svs files (3DHistech, Budapest, Hungary). The QuPath analysis encompasses a thorough evaluation of the hormone profile, the identification of CD34/MIB1-positive blood vessels, and an assessment of MIB1 for endothelial and tumor cells. Furthermore, the Allred score and H-score were evaluated in conjunction with the count of MIB1-positive nuclei. Analogous parameters were assessed using the same methodology in the neighboring tumor tissue of selected proliferating vessels to ascertain whether the tumor tissue exerts a significant influence on the proliferating endothelium. We imported all slides stained with CD34/MIB1 into QuPath version 0.4.3, an open-source platform for bioimage analysis of microscopic slides, where they were examined using integrated software and add-ons, such as Fiji and Vascular Analysis, for accurate evaluation of stromal blood vessels. QuPath is typically used in the literature to assess tumor stroma ratio, which seems to have a significant prognostic impact (Figure 1).

For each image, we set up H-DAB brightfield mode in QuPath. DIA started with a pre-processing step consisting of estimating stain vectors. We used image auto-calibration via the “Estimate Stain Vectors” function to correct for pixel depth variability between images. Briefly, we selected 6–20 regions of interest (ROIs) from stroma by using the brush tool, which gave us the most accurate delineation of stromal vessels from PitNET tissue. ROIs were defined as stromal vascular components which include blood vessels with positive signals for both MIB1 and CD34. We used the tumor versus stroma classifier related to MIB1 nuclear expression. Parameters for cell segmentation were focused on nuclear properties (size, intensity thresholds and smoothness). The analysis continued with cell detection by choosing a positive cell detection option and setting the cell and intensity parameters. Each detection image was assigned an optical density sum with a requested pixel size of 0.5 μm and cell expansion of 1.988 μm, including the nucleus. Intensity threshold parameters comprised the score compartment and three threshold levels were evaluated—weak (+1, highlighted with yellow), moderate (+2, highlighted with orange), and high (+3, highlighted in red)—with all cells selected in blue considered negative. When the automated system failed to count enough cells or detect positive cells, thresholds were changed, and the image was processed again using the updated thresholds. Validation of the positive threshold for each image was conducted through secondary evaluation of the annotations by a pathologist, both with and without highlights of positive and negative cells. During automated scoring, QuPath software (version 0.4.3) provided us with the cell count, percentage of positive and negative elements, and the density score and intensity score separately. Moreover, the H-score was included in the final evaluation conducted by QuPath.

Statistical analysis was conducted utilizing JAMOVI software version 3 (free and open-source, www.jamovi.org, accessed on 12 November 2025) employing descriptive statistical analysis, Pearson’s correlation test, paired sample *t*-test, and Student’s *t*-test, with significance established at *p* < 0.05. To determine if the proliferation index is associated with both compartments, we compared MIB1 levels (measured by QuPath analysis) in the tumor compartment to those in the endothelial compartment.

## 3. Results

We classified 109 PitNETs according to their hormone profile as follows: GH-secreting (*n* = 21), prolactin-secreting (PRL) (*n* = 30), thyroid-secreting (TSH) (*n* = 10), adrenocorticotropic (ACTH) (*n* = 11), follicle-stimulating (FSH) (*n* = 9) and luteinizing (LH) (*n* = 10). Eighteen cases were classified as non-functioning PitNETs. 

Descriptive statistics for the parameters used in this study are presented in Table 1.

**Figure 2:**
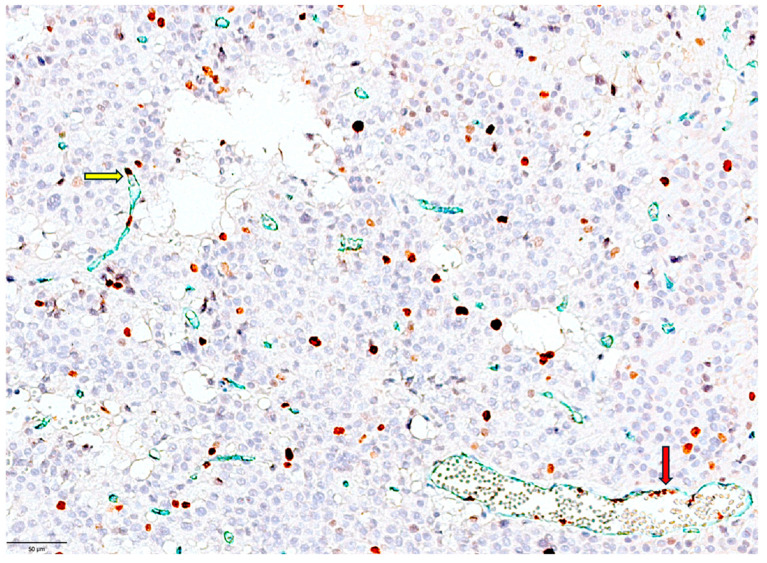
Double staining for CD34 (green)/MIB1 (brown) applied in PitNETs in our study aiming to identify the proliferative status of tumor cells (brown exclusively) and vascular endothelial cells (via colocalization of brown nuclear and green cytoplasm) on the same microscopic field in order to correlate the proliferative status from both compartments. PitNET vascular heterogeneity is illustrated by a mixture of small blood vessels (highlighted by CD34 staining in green, cytoplasmic) with positive nuclei (yellow arrow, brown staining with nuclear location) and mature functional vessels with a high number of proliferating endothelial cells lining the lumen (red arrow, brown staining with nuclear location). Microscopically, an increase in MIB1 LI in tumor cells surrounding proliferative vessels can be observed.

Endothelial MIB1LI had a heterogeneous distribution. MIB1 positivity was seen in tiny blood vessels (capillaries) and perfused blood vessels with well-defined lumens (Figure 2).

Normal pituitary tissue has a high MIB1 LI of capillary endothelial cell proliferation. A total of 98% of normal pituitary tissue has an Allred score of 5. This score stems from the high density (score of 4 QuPath analysis) and low intensity (score of 1 by QuPath scoring system) of MIB1-positive endothelial cells lining blood vessels. Conventional microscopy managed by human observation is unable to properly quantify low immunostaining intensities, whereas QuPath achieved this by combining density and intensity as the Allred score.

QuPAth also calculated the percentage of nuclear MIB1 LI in vascular regions of interest.

MIB1 was found in 63.34 percent of endothelial nuclei in normal pituitary tissue.

Hormone profile heterogeneity amongst the 109 PitNETs was high and had a strong impact on endothelial proliferation.

**Figure 3:**
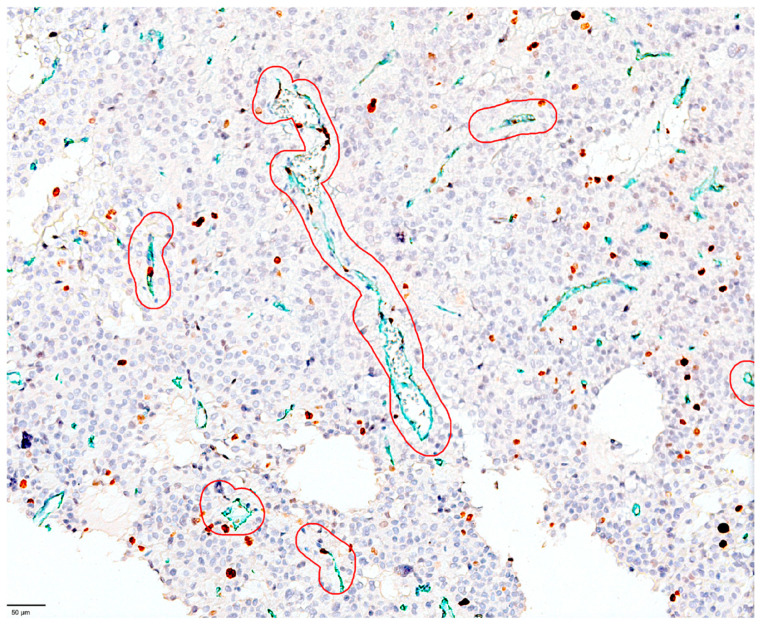
The identification and selection of proliferating vessels in the case of PRL-secreting PitNETs by using QuPath tools and analysis. Using the brush tool, we selected all vessels in which we identified colocalization of MIB1 LI (nuclear, brown) and CD34 (green, cytoplasmic). This case has an Allred score of 5 for proliferating endothelial cells and an Allred score of 4 for MIB1 LI in tumor cells surrounding proliferative blood vessels.

Regarding PRL secreting PitNETs, we observed that all cases had a high number of proliferating endothelial cells lining the tumor vasculature (Figure 3). Compared to other PitNET subtypes, we found that PRL secretion had the highest number of proliferating endothelial cells in the endothelium lining the blood vessels.

Regarding the other PitNET subtypes, high heterogeneity was observed for proliferating endothelial cells. Most cases of non-functioning PitNETs registered higher scores for proliferating endothelial cells compared to GH-secreting PitNETs. The lowest scores were observed for TSH-, ACTH-, and LH-secreting PitNETs for the endothelial compartment.

Non-functioning PitNETs had MIB1 LI at the endothelium level. Based on this microscopical evidence, we investigated whether endothelial cell proliferation and PitNET tumor cell proliferation are mutually influenced.

**Figure 4:**
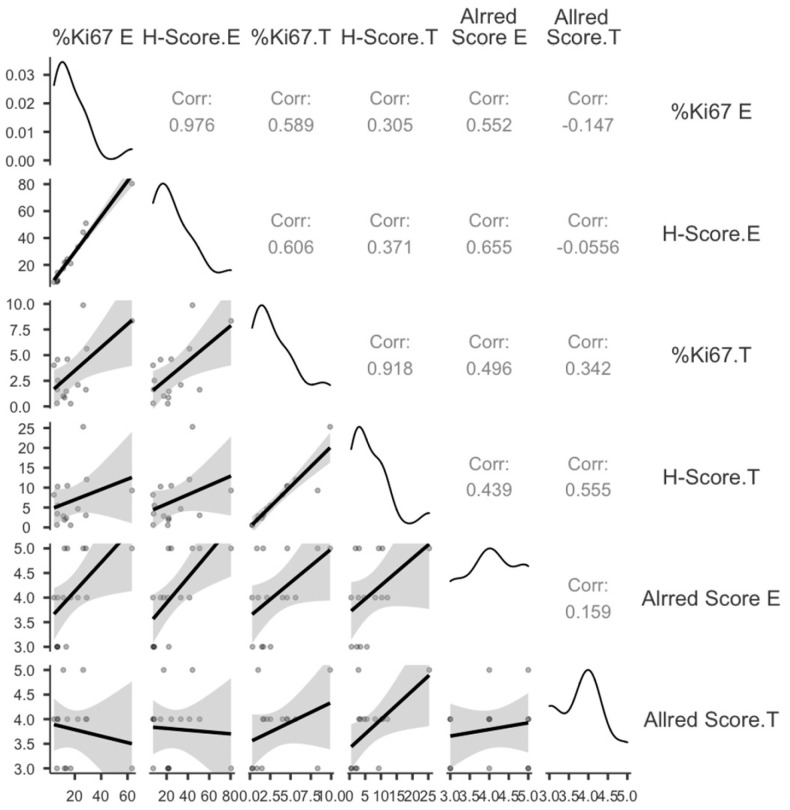
Correlation plot between parameters chosen to evaluate proliferation index in pituitary tumors. % Ki67 represents % MIB1 for both T (tumor cells) and E (endothelial cells).

PitNET-derived proliferating endothelial cells had an Allred score of 3–5. Moreover, 26.6% of endothelial cells had an Allred score of 3, 40% scored 4, and 33.3% scored 5. Although the Allred score for proliferating cells was between 3 and 5, 33.33% of cancer cells scored 3, 53.33% scored 4, and 15.34% scored 5. Table 2 summarizes the parameters used to compare tumor cell growth to endothelial cell proliferation. Statistical analysis shows that tumor and endothelial cell proliferation are interdependent. The percentage of MIB1 LI-positive tumor cells (%MIB1 LI.T) and endothelial cells (%MIB1 LI.E) in the same location correlated statistically (*p* = 0.01). The Allred score for endothelial cells correlated strongly with MIB1 LI.T % (*p* = 0.03). The proliferative status of endothelial and PitNET cells was significantly correlated (Table 2). This shows that proliferating tumor cells interact with proliferating endothelial cells. The results of the statistical analysis can be seen in Table 2 and Figure 4.

According to the results presented in Table 3, significant correlations were found to exist between MIB1 LI from proliferating tumor and stromal endothelial cells. Our findings provide evidence that tumor cells may exert an influence on blood vessel endothelial cells in pituitary adenomas with respect to proliferation.

**Figure 5:**
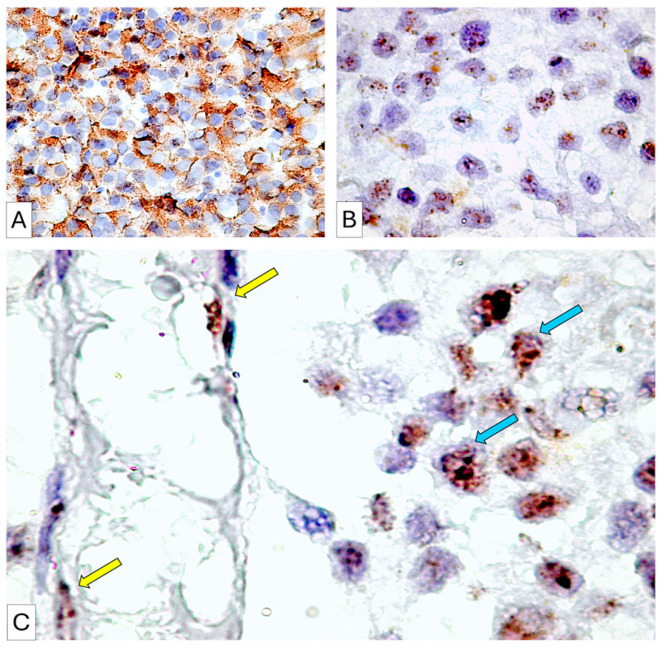
VEGF immunohistochemistry (**A**, cytoplasmic, granular, brown) and RNAscope (**B**,**C**, nuclear, dotted signals, for molecular validation) analyses in tumor cells of PRL-secreting PitNETs. Endothelial cells lining blood vessels in proximity to tumor areas showed high VEGF mRNA expression (**C**, yellow arrows). Notably, the intensity of the VEGF mRNA signal in these endothelial cells was comparable to that observed in adjacent tumor cells (**C**, blue arrows).

Based on this idea, we examined tumor cell VEGF expression and tumor proliferation. Each PitNETs hormonal subtype was analyzed independently. Both tumor and stromal endothelial cells expressed VEGF according to immunohistochemistry and RNAscope analyses (Figure 5A–C). The hormone profile of PitNET subtypes strongly influenced VEGF protein and molecular expression.

**Figure 6:**
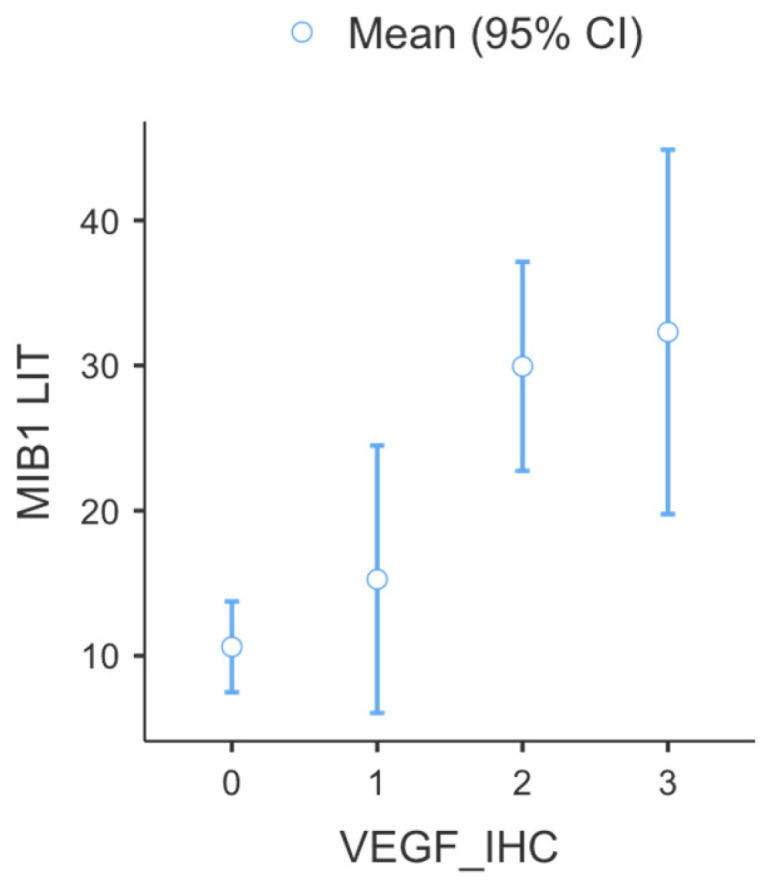
Graphic view of interrelation between VEGF expression and MIB1 index in tumor cells. Cases with VEGF scores of 2 or 3 had highest MIB1 index.

Descriptive statistical analysis of MIB1 related to VEGF expression was performed, and the results are detailed in Table 4 and Figure 6.

**Figure 7:**
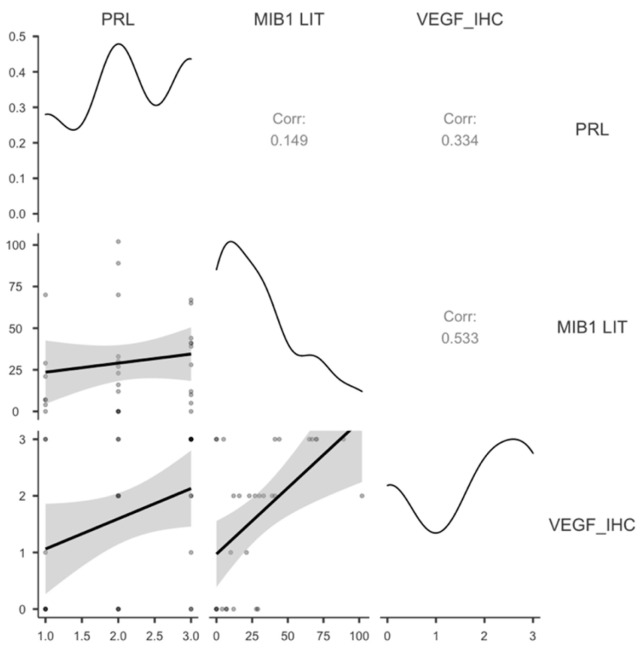
A scatter plot showing the significant correlations of VEGF expression in PRL-secreting PitNETs with MIB1 LIT. VEGF—Vascular Endothelial Growth Factor; MIB1 LIT—proliferation marker of MIB1 positivity in tumor cells.

Only PRL-secreting PitNETs showed a significant correlation between VEGF protein levels, PRL expression, and MIB1 LI-positive tumor cells (Table 5, Figure 7).

**Figure 8:**
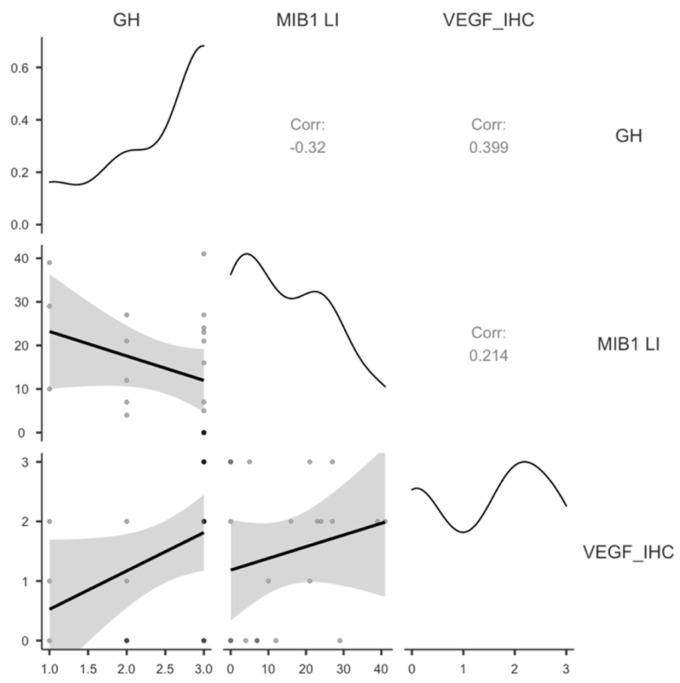
Scatter plot of statistical correlations found for GH-secreting PitNETs.

In GH-secreting PitNET cells, VEGF expression was significantly correlated with GH expression (*p =* 0.036) but not with MIB1 LI (*p* = 0.179) (Table 6, Figure 8).

**Figure 9:**
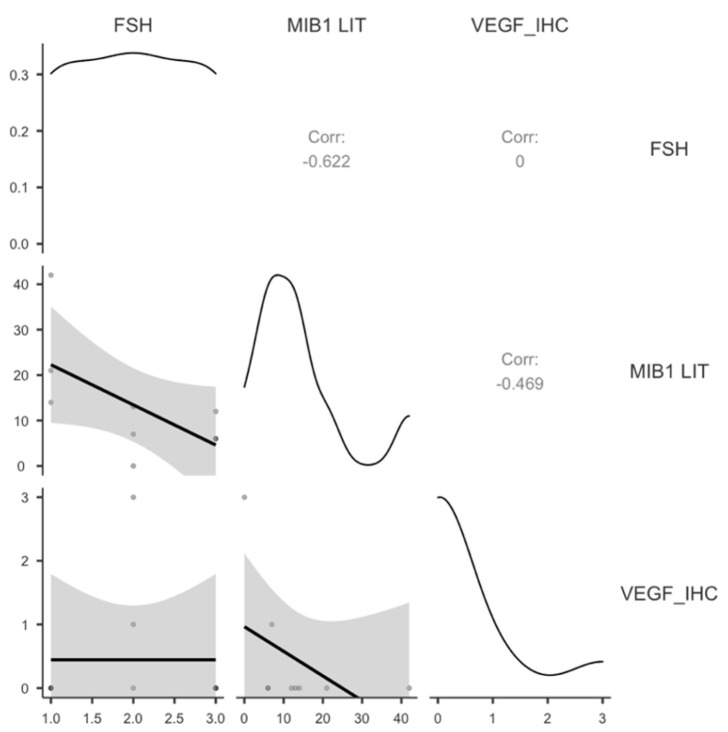
Graphic distribution of significant correlation for FSH-secreting PitNETs.

Proliferation of FSH-secreting PitNET cells was negatively correlated with FSH levels, while no correlation was found with VEGF levels (Table 7, Figure 9).

In TSH-, ACTH-, and LH-secreting PitNETs, tumor cell proliferation was not correlated with VEGF expression (*p* = 0.262, *p* = 0.745, and *p*= 0.250, respectively) nor with hormone levels (*p* = 0.181, *p* = 0.986, and *p* = 0.324, respectively).

**Figure 10:**
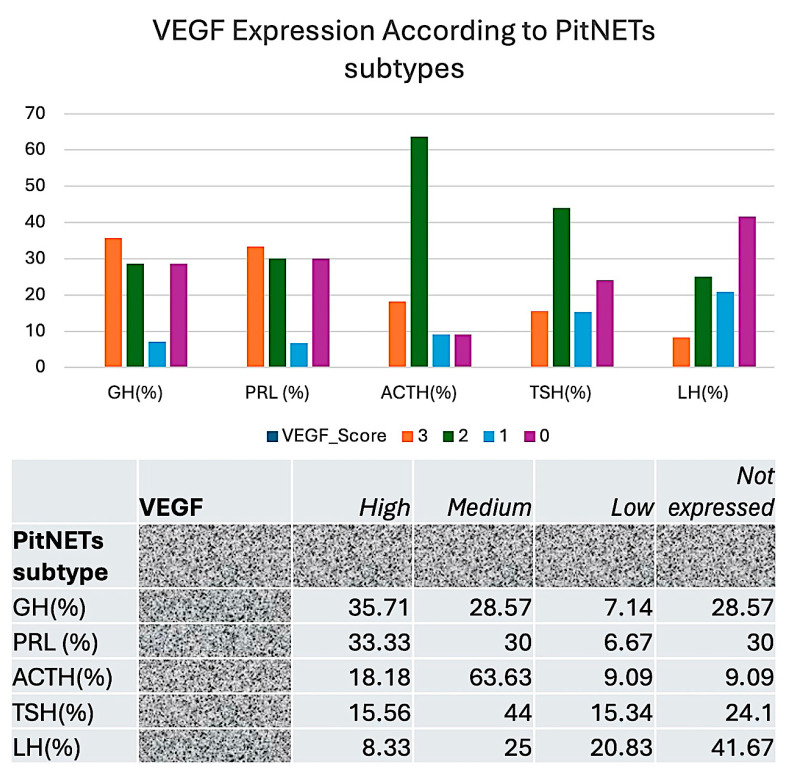
PitNETs subclasses of VEGF-expressing cases according to different PitNETs subtypes.

After we analyzed the correlation between MIB1 and VEGF for each PitNETs hormonal subtype, we aimed to classify and characterize these cases into distinct subclasses according to VEGF expression and MIB1 positivity due to a potential therapeutic impact related to anti-VEGF therapies. We defined three subclasses of VEGF-positive PitNETs: high, medium, and low VEGF-expressing PitNETs. Amongst different PitNETs subtypes, GH- and PRL-secreting PitNETs have the highest percentage of high VEGF-expressing cases (Figure 10).

**Figure 11:**
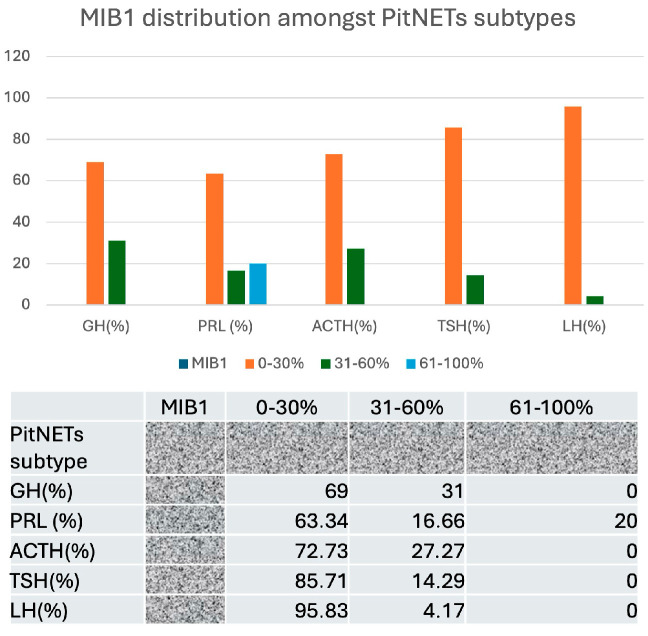
MIB1 LI distribution for different subtypes of PitNETs. Note that PRL-secreting PitNETs include about 20% of cases with a high proliferative index.

We also stratified PitNETs in terms of their ability to proliferate based on MIB1 LI. Once again, PRL-secreting PitNETs showed particular proliferative behavior, with about 20% of cases presenting a high proliferative index (61 to 100% out of total tumor cells) (Figure 11).

**Figure 12:**
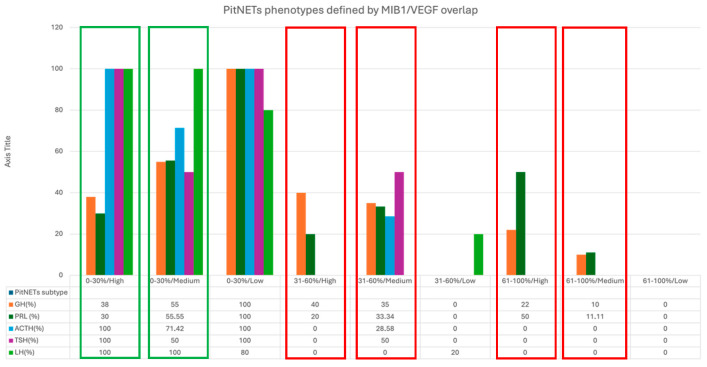
Overlapping MIB1 LI and VEGF expression patterns in distinct subgroups with different proliferation statuses and VEGF expression.

When we overlapped VEGF subgroups with MIB1_LI, we identified two different PitNETs subgroups: VEGF-dependent and VEGF-independent proliferative subgroups. The VEGF-dependent proliferative subgroup included GH- and PRL-secreting PitNETs, with high and/or medium VEGF expression correlated with an MIB1 LI between 31 and 100% (Figure 12, red quadrans). In the VEGF-independent subgroup, we found that the high and medium VEGF-expressing subgroup had an MIB1 LI ranging between 0 and 30% (Figure 12, green quadrans).

## 4. Discussion

The proliferative status of endothelial cells lining blood vessels from PitNETs remains one of the most controversial issues in the pathogenesis of PitNETs and angiogenesis research. For a long time, it has been accepted that the endothelium of normal human pituitary blood vessels has a higher proliferation rate compared to PitNETs, except for PRL-secreting and non-functioning PitNETs [19]. Previously, our team studied endothelial cell proliferation but in relation to microvessel density rather than performing a correlative assessment with the proliferation status of tumor cells [19]. Several mechanisms of angiogenesis in PitNETs have been previously described [9,20,21]. It seems that intussusceptive angiogenesis is specific to PRL secretion via an endoglin-mediated pathway, while sprouting angiogenesis occurs in other PitNETs. In a recent paper by Zhou et al., several genes encoding angiogenic growth factors were reported as key players in the angiogenic process. As VEGF family members, most of them induce endothelial cell proliferation and migration. Our previous studies already confirmed that angiogenic growth factors are highly expressed in PitNETs and may induce endothelial cell proliferation [22]. Angiogenic growth factors expression is highly dependent by hormone profile [22] but then we did not find any significant correlation in between them and endothelial and tumor cells proliferative status. The results from the present study partially overlap with those of our previous research, where we reported that VEGF_mRNA was upregulated in about 17% of PitNET cells, endothelial cells, and folliculo-stellate cells. It seems that angiogenic growth factors induce proliferation not only of endothelial cells but also of tumor cells due to autocrine and paracrine actions.

We highlighted here for the first time the mutual significant correlation between the proliferative status of tumor and endothelial cells from PitNET blood vessels by using digital image analysis software and QuPath analysis to conduct an automated evaluation, thus decreasing the subjectivity inherent in manual analysis.

Endothelial cell proliferation in PitNETs has not been directly discussed in previous papers due to the difficulty in assessing endothelial proliferation using conventional microscopic methods. The use of QuPath has not been reported before for the analysis of endothelial and tumor cell proliferation in human PitNETs. Scarce data have been reported on the use of QuPath for MIB1 LI assessment, and the existing data relate to mice pituitary tissue and not human PitNETs [23]. For human tissues, QuPath digital image analysis was applied mainly in neuroendocrine pituitary tumors for the assessment of Pit1 nuclear biomarkers on immunohistochemical stained slides [24]. By using manual quantification of MIB1 LI combined with artificial intelligence, Cai et al. developed a nomogram which can further assist neurosurgeons to develop better, more individualized treatment strategies for patients with PitNETs by predicting MIB1 LI preoperatively [25].

The invasive phenotype of PitNETs is highly dependent on angiogenesis according to recent data [26]. Li and his team revealed that HSPB1 is a common factor that promotes tumor and endothelial cell proliferation and migration through VEGF/VEGFR pathway activation [26]. In one of our previous works on growth factor expression in PitNETs, we found that Vascular Endothelial Growth Factor (VEGF) mRNA is overexpressed in both tumor and endothelial cells but also in folliculo-stellate cells from PitNETs, especially PRL- and GH-secreting PitNETs [22]. In this paper, we proved that VEGF-expressing tumor cells are highly proliferative compared to those which lack VEGF expression, with this process being dependent on hormone profile. It is widely recognized that VEGF is an angiogenic growth factor secreted by several tumor cell types (including those from PitNETs) with great potential for inducing endothelial cell proliferation and migration. Li et al. experimentally proved the mutual interrelation between gonadotropin-secreting GT1-1 cells and microvascular endothelial cells mediated by the HSPB1 gene. HSPB1 enhances the migration of bEnd.3 cells towards GT1-1 cells and facilitates the formation of blood vessels by bEnd.3 cells. bEnd.3 cells can release CCL3 and CCL4, thereby enhancing the vitality, proliferation, and migration of GT1-1 cells. HSPB1 enhances the angiogenesis of bEnd.3 cells, subsequently contributing to tumor growth in vivo, and serves as a critical gene in the invasion of the cavernous sinus in PitNETs, facilitating the remodeling of the tumor microenvironment by enhancing the formation of blood vessels in brain microvascular endothelial cells. The interaction between tumor cells and microvascular endothelial cells facilitates tumor progression. The authors suggested that the role of HSPB1 in facilitating tumor invasion through angiogenesis in PitNETs presents a potential target for therapeutic intervention in cases of PitNETs that invade the cavernous sinus. These findings are in line with our results. Our data compliment their experimental findings as they relate to human tissue specimens and they provide evidence of the mutual correlation between the proliferative status of tumor and endothelial cells in PRL-secreting and non-functioning PitNETs.

The strengths of the present study are as follows: This is the first report of the mutual association between the proliferation status of endothelial and tumor cells in PitNETs using AI-based tools for the evaluation of MIB1 LI in human PitNET tissue specimens. Moreover, we have demonstrated that the proliferative impact of VEGF is limited to PRL-secreting PitNETs.

The relatively small number of cases (109) could be considered a limitation of the present study. This is due to the fact that, currently, first-line therapy for PitNETs is drug-based rather than surgical. Our cases were retrospectively selected over a large period (15 years). Given the limited possibility to obtain human tissue fragments, we consider that 109 cases is a sufficient cohort for our research purpose, including statistical analysis.

Despite these limitations, our research could have deep clinical and therapeutic implications, especially in refractory and nonresponsive PitNETs cases. Due to the high proliferative index in both tumor and endothelial cells, PitNETs can grow faster and may experience rapid tumor expansion. The higher proliferation rate of PitNET endothelial cells induces the development of the neovasculature with high permeability and a lack of neovessel maturation and stabilization. This could lead to a higher risk of intratumoral hemorrhage or necrosis, presenting acutely with headache, visual loss, or hypopituitarism. A greater intraoperative bleeding risk during transsphenoidal/other resections may lengthen surgery, complicate visualization, and increase the risk of incomplete resection. A rich vascular/stromal microenvironment can restrict drug access or produce resistance niches.

High PitNETs vascularization should be highlighted in histopathology reports and/or imaging reports and may be considered by clinicians a “red flag” indicating a potentially more aggressive tumor phenotype. For refractory/aggressive tumors, molecular testing should be discussed by an interdisciplinary team (endocrinologist, pathologist, genetician, surgeon and radiologist), who may opt for temozolomide use. Potential enrollment in trials of antiangiogenic or combined therapies should be recommended.

## 5. Conclusions

Using QuPath digital image analysis, our findings show the reciprocal relationship between proliferative tumor cells and proliferative endothelium cells in PitNETs. This MIB1 LI-mediated reciprocal tumor stroma contact is unique to PRL-secreting and non-functioning PitNETs. This finding may point to the existence of multiple factors that can frequently cause endothelial cells to become both activating and proliferating, as well as leading to the proliferation of PitNETs cells. This, in turn, may support a higher level of aggression of PitNETs or the occurrence of therapy resistance.

## Figures and Tables

**Table 1:** Descriptives statistical analysis of the parameters included in the present study.

	N	Mean	Median	SD	SE
% MIB1_E	109	17.77	13.45	14.489	1.3878
H-Score.E	109	26.67	21.78	19.701	1.8870
%MIB1_T	109	3.18	2.10	2.792	0.2674
H-Score.T	109	6.54	4.66	6.144	0.5885
Allred Score E	109	4.05	4	0.786	0.0753
Allred Score.T	109	3.79	4	0.654	0.0626

Abbreviations: H-score in tumor (T) and endothelial cells (E) provides continuous, quantitative scale (0–300) to represent protein expression levels. H-scores are used in digital pathology for diagnosis, predicting patient prognosis, and guiding therapeutic decisions. Allred score defines both density and intensity of MIB1 immunopositive proliferating nuclei. Number of samples (N = 109).

**Table 2:** Correlation matrix between PitNETs cell parameters and endothelial cells from tumor blood vessels.

		% MIB1 LI.E	H-Score. E	% MIB1 LI.T
% MIB1 LI.T	Pearson’s r	0.589 *	0.606 **	—
	*p*-value	**0.010**	**0.008**	—
Allred Score E	Pearson’s r	0.552 *	0.655 **	0.496 *
	*p*-value	0.016	0.004	**0.030**
Allred Score.T	Pearson’s r	−0.147	−0.056	0.342
	*p*-value	0.699	0.578	0.106

Note. * *p* < 0.05, ** *p* < 0.01; one-tailed.

**Table 3:** Paired samples *t*-test for parameters selected to evaluate proliferative index in PitNET and stromal endothelial compartments.

Paired Samples *t*-Test					
			Statistic	df	*p*
% MIB1 LI.E	H-Score.E	Student’s *t*	−5.306	108.0	<0.001
	% MIB1 LI.T	Student’s *t*	4.184	108.0	< 0.001
	H-Score.T	Student’s *t*	3.003	108.0	0.009
	Allred Score E	Student’s *t*	3.661	108.0	0.003
	Allred Score.T	Student’s *t*	3.599	108.0	0.003
H-Score.E	% MIB1 LI.T	Student’s *t*	4.878	108.0	<0.001
	H-Score.T	Student’s *t*	4.121	108.0	0.001
	Allred Score E	Student’s *t*	4.448	108.0	<0.001
	Allred Score.T	Student’s *t*	4.376	108.0	<0.001
% MIB1 LI.T	H-Score.T	Student’s *t*	−3.421	108.0	0.004
	Allred Score E	Student’s *t*	−1.208	108.0	0.247
	Allred Score.T	Student’s *t*	−0.768	108.0	0.455
H-Score.T	Allred Score E	Student’s *t*	1.677	108.0	0.116
	Allred Score.T	Student’s *t*	1.859	108.0	0.084

Abbreviations: % MIB1 LI.E—percentage of proliferative endothelial cells; % MIB1 LI.T—percentage of proliferative tumor cells. H-score in tumor (T) and endothelial cells (E) provides continuous, quantitative scale (0–300) to represent protein expression levels. H-scores are used in digital pathology for diagnosis, predicting patient prognosis, and guiding therapeutic decisions. Allred score defines both density and intensity of MIB1 immunopositive proliferating nuclei. Number of samples (N = 109).

**Table 4:** Standard deviation (SD), standard error (SE) and mean calculated for interrelation between VEGF and MIB1 expression in PitNETs.

	VEGF_IHC	N	Mean	SD	SE
MIB1 LIT	0	34	10.6	8.97	1.54
	1	11	15.3	13.71	4.13
	2	35	29.9	20.96	3.54
	3	29	32.3	33.01	6.13

**Table 5:** Pearson’s correlation for VEGF with PRL expression and MIB1 LI in tumor cells.

		PRL	MIB1 LIT
VEGF_IHC	Pearson’s r	0.334 *	0.533 **
	*p*-value	0.036	0.001
	N	30	30

Note. * *p* < 0.05, ** *p* < 0.01; one-tailed. PRL—prolactin; VEGF_IHC—Vascular Endothelial Growth Factor protein expression assessed by immunohistochemistry; MIB1 LIT—proliferation marker of MIB1 positivity in tumor cells.

**Table 6:** Significant correlation between GH and VEGF in GH-secreting PitNETs.

		GH	MIB1 LI
MIB1 LI	Pearson’s r	−0.320	—
	*p*-value	0.921	—
	N	21	—
VEGF_IHC	Pearson’s r	0.399 *	0.214
	*p*-value	0.036	0.176
	N	21	21

GH—growth hormone; VEGF_IHC—Vascular Endothelial Growth Factor protein expression assessed by immunohistochemistry; MIB1 LI—proliferation marker of MIB1 positivity in tumor cells. Note. * *p* < 0.05; one-tailed.

**Table 7:** FSH is inversely significantly correlated with MIB1 LI but not with VEGF protein expression (VEGF_IHC).

		FSH	MIB1 LIT
MIB1 LIT	Pearson’s r	−0.622 *	—
	*p*-value	0.037	—
	N	9	—
VEGF_IHC	Pearson’s r	0.000	−0.469
	*p*-value	0.500	0.101
	N	9	9

FSH—follicle-stimulating hormone; VEGF_IHC—Vascular Endothelial Growth Factor protein expression assessed by immunohistochemistry; MIB1 LIT—marker of MIB1 positivity in tumor cells. Note. * *p* < 0.05; one-tailed.

## Data Availability

The original contributions presented in this study are included in the article. Further inquiries can be directed to the corresponding authors.
